# Seroprevalence and risk factors for *toxoplasma *infection among pregnant women in Aydin province, Turkey

**DOI:** 10.1186/1471-2458-5-66

**Published:** 2005-06-15

**Authors:** Sema Ertug, Pinar Okyay, Munevver Turkmen, Hasan Yuksel

**Affiliations:** 1 Department of Parasitology, Adnan Menderes University, School of Medicine, Aydin, Turkey; 2 Department of Public Health, Adnan Menderes University, School of Medicine, Aydin, Turkey; 3 Department of Child Health, Adnan Menderes University, School of Medicine, Aydin, Turkey; 4 Department of Gynecology, Adnan Menderes University, School of Medicine, Aydin, Turkey

## Abstract

**Background:**

The aims of the present study were to determine the prevalence of toxoplasmosis in pregnant women at first trimester of their pregnancy and to follow up the seroconversion for next two trimesters, and to identify the risk factors and possible contamination routes in Aydin province, Turkey.

**Method:**

The sample size was calculated as 423 on a prevalence of 50%, d=0.05 at a confidence level of 95% with 10% addition. It was a cross-sectional study with multistage sampling. After a questionnaire applied to the pregnant women, anti-Toxoplasma IgG antibodies were studied with ELISA and IFA, values in conflict with DA test, where IgM antibodies were studied with ELISA and for borderline or positive values of IgM avidity test was used.

**Results:**

The mean age of 389 (92.9%) of pregnant women in the study was 24.28+/-4.56 years, the seroprevalence of anti-Toxoplasma IgG antibodies for toxoplasmosis was 30.1%. Seroprevalence was increased with age (p=0.001) and with drinking water consumption other than bottled water (p=0.042). No significant relations were observed between anti-Toxoplasma IgG antibodies and education level, being native or migrant, abortion history, consumption of meat, vegetable and milk/milk products, personal or kitchen hygiene habits, cat owning at home of the pregnant women. No IgM antibody was detected.

**Conclusion:**

One of every three pregnant women in Aydin was at risk of toxoplasmosis at the first trimester of their pregnancy. Increased seroprevalance with age was a predictable result because of increasing time of exposure. Increased seroprevalence with consumption of municipal and uncontrolled water (well/spring water) supplies was similar with latest epidemiological findings.

## Background

*Toxoplasma gondii (T.gondii)*, an obligate intracellular parasite found in many species throughout the world, causes a variety of clinical syndromes in human and animals [1]. Toxoplasmosis during pregnancy can cause congenital infection and manifest as mental retardation and blindness in the infant. The severity of fetal disease varies inversely with the gestational age at which maternal infection occurs [2].

Seroprevalence estimated for human population varies greatly among different countries, among different geographical areas within one country, and among different ethnic groups living in the same area [2]. Seroprevalence of *T.gondii *infection in women at childbearing age is found to be between 4%-100%. Incidence of primary maternal infection during pregnancy varies in a range of 1 to 310 per 10.000 pregnancies in the populations in Europe, Asia, Australia, and the Americas [2]. The serological screening of pregnant women for toxoplasmosis and the follow-up until delivery are not routine procedures in Turkey. In a few studies performed in our country, seroprevalence of *T.gondii *infection in women at childbearing age is found to be between 19.2% to 85%; and it is estimated that incidence of congenital toxoplasmosis is 0.1% [3-5].

Major routes of infection are: a) ingestion of oocysts through close contact with infected cat or cat's faeces, b) ingestion of water or food contaminated with the oocysts, c) eating raw or undercooked meat from infected animals that contain the tissue cysts, d) transplantation of infected organs, and e) congenital infection [1]. The following risk factors have also been identified in recent epidemiological studies: owning cats [6], eating raw or uncooked pork, lamb, mutton, beef, game or mincemeat products [6-10], eating raw or unwashed vegetables or fruits [8], frequent consumption of raw vegetables outside the home [6], travelling outside of Europe, the United States and Canada [7], having poor hand hygiene [6], washing kitchen knives infrequently [8], cleaning the cat litter box [8], contact with soil [7], a history of working in soil-related occupations [11]. Similarly, outbreaks of toxoplasmosis have been reported to be related with eating raw or uncooked pork, lamb, mutton or beef products [12] and additionally, with oocyst contaminated water [13] or soil [14].

The aims of the present study were to determine the prevalence of toxoplasmosis in pregnant women at the first trimester of their pregnancy and to follow up the seroconversion for the next two trimesters, and to identify the risk factors and possible contamination routes in the Aydin province, Turkey.

## Methods

### Study population

The study was performed in Aydin, one of the major cities in the Aegean region of Turkey with 8.007 km^2 ^and 903677 population in 2004. Apart from the industry, the main agricultural products of the province are figs, olives, strawberries and cotton; especially figs are known throughout the world. The ancient name of the province of Aydin was Tralleis. It was celebrated as the center of sculpture, with a well known sculpture school. The data was acquired from health centers in urban and rural areas of Aydin. The study design was cross-sectional. The sample size was calculated as 384 on a prevalence of 50%, d = 0.05 at a confidence level of 95%. A total of 10% of the sample population was added to the sample size; so, the final study population size was 423. Multistage sampling was used in the selection of the study group. Aydin was separated into four regions according to the socio-economic and health data taken from Directory of Health. Four health centers (Two urban and two rural) were randomly selected from each region. The pregnant women were admitted into the study at their first visit to health center or when first detected as pregnant in the field by the midwife responsible for the selected population (approximately 2500 people per midwife) during a six month period. Permission was taken from Directorate of Health. A questionnaire was performed. There were questions eliciting socio-demographic data including age, education (*illiteracy, primary school, high school, university or more*), occupation, parity, residency and related risk factors including source of drinking water (*general network, bottled water, well, spring, tap water, other*), obstetrical history (*total number of pregnancy, stillbirths, abortions and live births*), frequency (*one meal a day, a few meals in a week, a few meals in a month, seldom/never*) and type of meat (*beef, lamb, chicken, game, pork, delicatessen*), vegetables and fruits (*raw or not, at home or outside*), egg, mayonnaise and milk (*pasteurised, unpasteurised*) consumption, cooking preferences (*raw, rare-if the center is still raw, medium-if the center is still pink, well-done-if no pink meat is seen*), kitchen hygiene (*hand washing/kitchen utensils infrequently*), owning cat (*outdoor or indoo*r), history of cleaning cat litter box or feeding raw meat scraps, eating out (*never, frequency- often: at least once a week, sometimes: a few a months, rare: a few in a year*), travelling abroad, soil exposure (*occupation or hobby*). Informed consent was obtained from the participant.

### Serum samples

In the first trimester, 389 (92.0%) women could be reached. During follow-up period, 257 (66.1 %) of them in the second trimester and 124 (31.9%) of them in the third trimester could be reached. Only one serum sample for each pregnant woman was studied at each trimester, so a total of 770 serum samples were studied at the end of the study.

Each blood sample was taken by a nurse at the health center and then centrifuged to separate serum and kept at -20°C in the laboratory of the department of parasitology. Serological study was performed within one week.

### Serological methods

The *Toxoplasma *specific IgG antibodies in 770 serum samples of three trimesters were studied by Enzyme Linked Immunosorbant Assay (ELISA) and methods. In the first trimester (n = 389), there was a disagreement between ELISA and Indirect Immunofluorescence Antibody (IFA) methods (IFA negative, ELISA positive) in six (1.54%) serum samples, then these samples were also studied by direct agglutination test (DA) and no antibody answer was observed. No disagreement between ELISA and IFA methods was observed in other samples.

The *Toxoplasma *specific IgM antibodies were studied for 770 serum samples of three trimesters by ELISA method. Ten border results (1.30%) and three positive results (0.39%) were also assessed by avidity test. There was high avidity at 13 (1.69%) serum samples.

#### a. ELISA

The *Toxoplasma *specific IgG antibodies were studied by commercial kit (Biokit-Bioelisa Toxo IgG/Italy) according to the manufacturer's instructions. All specimens were analysed using enzyme immunoassay test. The results more then 10 IU/ml were taken as positive results in the current study. The consistency between IFAT and bioELISA IgG kit was found high in an earlier study performed in the parasitology laboratory (kappa = 0.941, p = 0000) (15).

The *Toxoplasma *specific IgM antibodies were studied by commercial kit (Organon-Toxonostika IgM II MikroELISA kit) according to the manufacturer's instructions. The consistency of this commercial kit was found to be high by comparisons with many other commercial kits [16].

#### b. IFA test

Antigen preparations were made from tachyzoites of the TRH strain of *T.gondii*. Tachyzoites were obtained from the peritoneal exudates of mice infected 2 days earlier. These antigens have been routinely used for the serological tests in our laboratory [17]. Dilutions of 1:16 and higher were evaluated as positive in the current study.

#### c. DA test

Serum samples were analysed for antibodies to *T. gondii *by the direct agglutination test using formalin fixed tachyzoites as antigen. Titers ≥ 20 were considered positive [18,19].

#### d. Avidity Test

EIAgen Toxoplasma IgG Avidity test by Adaltis (Italy) was used in the study and avidity was considered as high avidity for results more then 35%, as low avidity for less then 30% and borderline avidity between 30%-35%.

### Statistical assessment

A statistical software package was used for data analysis. The descriptive data was given as mean ± standard deviation (SD). The chi-squared test was used for the analytic assessment. The differences were considered to be statistically significant when the p value obtained was less than 0.05.

## Results

There were 389 pregnant women at the beginning of the study. The mean age of the women was 24.28 ± 4.56 years. The mean gestation week was 9.85 ± 2.21. The means of total number of pregnancies, total abortion and stillbirth, and total live births were 2.01 ± 1.37, 0.36 ± 0.80 and 0.65 ± 0.82, respectively.

The seroprevalence of *Toxoplasma *specific IgG was 30.1% among pregnant women at the first trimester of their pregnancy (n = 389). During the follow-up during pregnancy, we could reach 257 (66.1 %) women in the second trimester and 124 (31.9%) women in the third trimester. No *Toxoplasma *specific IgM in total 770 serum samples of three trimesters was detected.

Socio-demographic characteristics and obstetrical history are given in Table 1. Risk factors related to eating preferences and hygienic habits are given in Table 2.

**Table 1:** Toxoplasmosis and sociodemographic/obstetric factors in the study population

**Risk factors**	**Toxoplasma IgG**				
		
	Negative		Positive		
		
	n	%	n	%	p
**Sociodemographic factors**					
Age (n = 356)					
15–29	223	72.2	86	27.8	**0.001**
30–40	23	48.9	24	51.1	
Education (n = 357)					
Less than primary school	171	69.0	77	31.0	0.978
Secondary school and more	75	68.8	34	31.2	
Occupation (n = 358)					
Housewife	230	68.9	104	31.1	0.840
Other	17	70.8	7	29.2	
Residence (n = 389)					
Urban	218	68.6	100	31.4	0.213
Rural	54	76.1	17	23.9	
**Environmental factors**					
Drinking water (n = 358)					
General network	136	67.0	67	33.0	**0.042**
Bottled	43	84.3	8	15.7	
Other	69	66.3	110	30.7	
**Obstetric history**					
Abortion history (n = 357)					
None	189	70.8	78	29.2	0.186
One or more	57	63.3	33	36.7	

**Table 2:** Toxoplasmosis and risk factors related to eating preferences and hygienic habits.

**Risk factors**	Toxoplasma IgG				
		
	Negative		Positive		
		
	n	%	n	%	p
Frequency of meat consumption (n = 338)					
Every day	12	54.5	10	45.5	0.099
A few times per week or less	225	71.2	91	28.8	
Cooking preferences (n = 333)					
Undercooked	38	66.7	19	33.3	0.707
Well-done	191	69.2	85	30.8	
Eating raw meat* (n = 341)					
Yes	32	68.9	13	28.9	0.767
No	204	68.9	92	31.1	
Type of meat					
Beef (n = 330)					
Yes	181	68.8	82	31.2	0.493
No	49	73.1	18	26.9	
Poultry(n = 352)					
Yes	233	69.1	104	30.9	0.839
No	10	66.7	5	33.3	
Game(n = 301)					
Yes	27	84.4	5	15.6	0.080
No	187	69.5	82	30.5	
Delicatessen (n = 340)					
Yes	162	68.9	73	31.1	0.522
No	76	72.4	29	27.6	
Milk and milk products(n = 355)					
Yes	193	69.9	83	30.1	0.958
No	55	69.6	24	30.4	
Eating raw vegetables					
Yes	235	69.5	103	30.5	0.982
No	9	69.2	4	30.6	
Washing kitchen utensils after cutting vegetables (n = 345)					
Yes	241	70.5	101	29.5	0.886
No	2	66.7	1	33.3	
Washing hands before meals (n = 351)					
Yes	239	68.9	108	31.1	0.180
No	4	100.0	0	-	
Eating outside of the home					
Yes	141	66.8	70	33.2	0.245
No	106	72.6	40	27.4	
Current cat ownership(n = 353)					
Yes	5	62.5	3	37.5	0.695
No	238	69.9	107	31.0	

• A Turkish delicacy made of spiced raw meat

## Discussion

Seroprevalence of *T.gondii *infection range between 15%-77% in different countries [9,11]. In the current study, the seroprevalence of *Toxoplasma *specific IgG was 30.1% in pregnant women at the first trimester. In outpatient women at childbearing age it is found to be between 21.8% to 85% in our country [4,20-23]. The only population-based study that could be reached was performed in a village. In that study, the seroprevalence of toxoplasmosis was found to be 30% in 600 people aged 7–50 years [24]. Latter seroprevalence is similar with the current study.

As in the study of Bobic et al. (1998) that have found that prevalence increases as the age increases [9], age effect was observed in the current study. The reason might be increasing risk of exposure with age.

In the current study, no statistical meaningful difference was observed between urban and rural areas for toxoplasmosis seroprevalence. Baril et al.(1999) [6] have found similar results with the current study; however, Ades et al (1993) [25] have found higher seroprevalences in urban areas. There is a need to assess the nature of infection chain for Aydin.

Seroprevalence was found to be changed according to usage of different drinking water sources in the current study. The highest prevalence (33.7%) was in the people using general network water, followed by uncontrolled water sources (30.7%). The municipal network water in Aydın is collected to processing pools from open springs next to a few villages. The high seroprevalence in general network water users may be in accordance with the latest articles that have showed the presence of oocysts in chlorinated network water. A study is needed to investigate oocysts in domestic and outdoor life for those whose water sources are the same. Lower prevalences in bottled water users might be explained in that the containers are filled soon after the water surfaces. Oocyst form of *T.gondii *seems to be the major factor in the infection of water resources. Bowie et al (1997) assessed an outbreak in the western Canadian province of British Columbia and found that a municipal water system that used unfiltered, chloraminated surface water had been the likely cause of the large community-wide outbreak of toxoplasmosis [26].

Frequent consumption and type of meat (pig, sheep and goat) were identified as the principle risk factor in several recent studies of *T.gondii *infections in humans [6-9,27]. No relation was observed between seroprevalence and type of meat consumed in the current study. In Turkey, beef and lamb are commonly used, especially mixed together. Pork is never used due to religious ban. However, it is known that pork is mixed with other type of meat and served without knowledge of consumers.

The cooking temperature of meat is an important issue in the infection of *T.gondii*. Thorough cooking is always preferred in Turkey. However, raw meat is also consumed in traditional cuisine as "cig kofte" which is a mixture of raw meats. In the current study, no statistical difference was observed between *T.gondii *prevalence, and cooking preferences and consumption of raw meat.

The association of cats and human toxoplasmosis is difficult to assess by epidemiological surveys because soil, not the cats, is the main culprit. Oocysts are not found on cat fur [28] and are often buried in soil along with cat faeces, and soil contact is universal and difficult to avoid [29]. In Turkey, cats and dogs are commonly stray animals. There is always a possibility that oocysts of the stray cats may gain infectious ability outside the animal. In the current study, the number of cat owners was just a few, and no relation was detected.

In the previous studies, lower educational level, soil-related occupations [11], eating raw or unwashed vegetables or fruits, cleaning the cat litter box [8], having poor hand hygiene [6,8], eating raw vegetables outside the home [6], contact with soil [7], travelling outside of Europe, the United States and Canada [7] were all found as risk factors for toxoplasmosis. These factors were assessed in the current study; however, no relation was found.

The importance of the current study is that it is one of the few – to our knowledge only one -population based studies performed in Turkey. However, there are some restrictions in the study. First of all, dye test could not be used as a gold standard. In order to overcome this restriction, different test were used together. Secondly, although health centers as primary care units cover all the population which they are responsible for, and the data obtained from them is the most appropriate for population-based studies, there may be a few women that could not be reached. Maybe one of the most important restrictions is that the assessment of risk factors was done according to information given by the patient. Some issues such as hygienic behaviour might be misrepresented as higher because of shaming or other factors. However, an improvement in the quality of data was attempted by using district midwifes who were familiar and trusted by the women during data collection.

## Conclusion

In conclusion, 69.9 % of pregnant women in the first trimester in the Aydin Province-Turkey, are susceptible to acute infection, and no *Toxoplasma *specific IgM was detected during the follow-up of pregnant women for three trimesters. This may be due to lower ratios of serum assessment in the second (66.1 %) and in the third (31.9%) trimesters. At the current moment, there is no legal obligation for education about means of minimizing exposure to *T.gondii*. in our country. Health authorities, especially primary health care givers should be sensitive to the importance of the issue. Hygiene of water supplies is also important. Future studies on possibility of contamination of network water by oocysts should be performed and used for prevention activities. The data found in the current study point out that due to a high risk of toxoplasmosis in pregnant women, a debate on developing a general screening program for toxoplasmosis in pregnancy in Turkey should be begun.

## Competing interests

The author(s) declare that they have no competing interests.

## Authors' contributions

SE planned the research; performed parasitic examinations and contributed discussing the results and writing manuscript. PO performed the sampling and statistical analyzes and contributed discussing the results and writing manuscript. MT participated in initial study design, and revised the manuscript. HY participated in initial study design, and revised the manuscript. All authors read and approved the final manuscript.

## Pre-publication history

The pre-publication history for this paper can be accessed here:


